# Chinese expert consensus on interventional diagnosis and management of acquired digestive‐respiratory tract fistulas (second edition)

**DOI:** 10.1111/crj.13607

**Published:** 2023-04-24

**Authors:** Hongwu Wang, Wen Li, Zikai Wang, Liangan Chen, Guoxiang Lai, Faguang Jin, Mingyao Ke, Jiayuan Sun, Jie Zhang, Baosong Xie, Nan Zhang, Wangping Li, Hongmei Zhou, Xiaoping Wang, Dianjie Lin, Yunzhi Zhou, Huaping Zhang, Dongmei Li, Changhui Wang, Xiaolian Song, Juan Wang, Shiman Wu, Junyong Yang, Lei Zhang, Meimei Tao, Yiming Zeng, Heng Zou, Hui Li, Fujie Song, Zhengbu Sha, Qiang Tan, Minghua Cong, Hanping Shi, Zhina Wang, Xinwei Han, Lingfei Luo, Hongming Ma, Gang Wu, Zhiqiang Wang, Xiaochuan Liu, Weiping Wu, Lishan Zhang, Yongan Ye, Guangying Zhu

**Affiliations:** ^1^ Center for Respiratory Disease, Dongzhimen Hospital Beijing University of Chinese Medicine Beijing China; ^2^ Department of Respiratory Medicine PLA General Hospital Beijing China; ^3^ Department of Respiratory Medicine 900 Hospital of the Joint Logistics Support Force of the Chinese People's Liberation Army Fuzhou China; ^4^ Department of Respiratory Medicine, Tangdu Hospital Air Force Medical University China; ^5^ Department of Respiratory Medicine Xiamen Second Hospital Xiamen China; ^6^ Department of Respiratory Medicine Shanghai Chest Hospital Shanghai China; ^7^ Department of Respiratory Medicine, Beijing Tiantan Hospital Capital Medical University Beijing China; ^8^ Department of Respiratory Medicine Fujian Provincial Hospital Fuzhou China; ^9^ Department of Respiratory Medicine Emergency General Hospital Beijing China; ^10^ Department of Respiratory Medicine Tianyou Hospital Affiliated to Tongji University Shanghai China; ^11^ Department of Respiratory Medicine Shandong Provincial Chest Hospital Jinan China; ^12^ Department of Respiratory Medicine Shandong Provincial Hospital Jinan China; ^13^ Department of Respiratory Medicine The Second Affiliated Hospital of Fujian Medical University Quanzhou China; ^14^ Department of Respiratory Medicine Shanghai Tenth People's Hospital Shanghai China; ^15^ Department of Respiratory Medicine The First Affiliated Hospital of Shanxi Medical University Taiyuan China; ^16^ Department of Respiratory Medicine Xinjiang Chest Hospital Wulumuqi China; ^17^ Department of Thoracic Surgery Beijing Chaoyang Hospital Affiliated to Capital Medical University Beijing China; ^18^ Department of Thoracic Surgery, Dongzhimen Hospital Beijing University of Chinese Medicine Beijing China; ^19^ Department of Thoracic Surgery The Third Affiliated Hospital of Xuzhou Medical University China; ^20^ Department of Thoracic Surgery Shanghai Lung Hospital Shanghai China; ^21^ Department of Oncology Cancer Hospital of Chinese Academy of Medical Sciences Beijing China; ^22^ Department of Oncology Beijing Shijitan Hospital Affiliated to Capital Medical University Beijing China; ^23^ Department of Oncology Emergency General Hospital Beijing China; ^24^ Department of Interventional Radiology The First Affiliated Hospital of Zhengzhou Medical University Zhengzhou China; ^25^ Department of Interventional Radiology Emergency General Hospital Beijing China; ^26^ Department of Gastroenterology PLA General Hospital Beijing China; ^27^ Department of Gastroenterology Emergency General Hospital Beijing China; ^28^ Department of Gastroenterology, Dongzhimen Hospital Beijing University of Chinese Medicine Beijing China; ^29^ Department of Radiology Beijing Cancer Hospital Affiliated to Peking University Beijing China

**Keywords:** acquired digestive‐respiratory tract fistulas, consensus, diagnosis, interventional therapy, management

## Abstract

Acquired digestive‐respiratory tract fistulas occur with abnormal communication between the respiratory tract and digestive tract caused by a variety of benign or malignant diseases, leading to the alimentary canal contents in the respiratory tract. Although various departments have been actively exploring advanced fistula closure techniques, including surgical methods and multimodal therapy, some of which have gotten good clinical effects, there are few large‐scale evidence‐based medical data to guide clinical diagnosis and treatment. The guidelines update the etiology, classification, pathogenesis, diagnosis, and management of acquired digestive‐respiratory tract fistulas. It has been proved that the implantation of the respiratory and digestive stent is the most important and best treatment for acquired digestive‐respiratory tract fistulas. The guidelines conduct an in‐depth review of the current evidence and introduce in detail the selection of stents, implantation methods, postoperative management and efficacy evaluation.

## 1 INTRODUCTION

Airway fistulas are defined as the loss of airway wall integrity and the formation of a fistula due to various reasons. Suppose the fistula causes the communication between the respiratory tract and the digestive tract, which is known as the digestive‐respiratory tract fistula, leading to the alimentary canal contents into the respiratory tract. In that case, it causes cough, fever, expectoration, and other respiratory symptoms and is a critical disease concern by many disciplines, such as the respiratory department, gastroenterology department, thoracic surgery department, interventional department, radiology department and oncology department.

According to the etiology, it can be divided into congenital and secondary. This consensus only discusses acquired fistula. The first edition of “expert consensus on diagnosis and management of acquired respiratory‐digestive tract fistulas” (including Chinese and English) has received extensive clinical attention since it was published in 2018 and has played a great role in standardizing the diagnosis and management of the disease. At the same time, many colleagues have also put forward valuable suggestions.

In recent years, various departments have been actively exploring various fistula closure techniques, some of which have been very successful, effectively improving the quality of patients' life and prolonging survival time. However, there is still a lack of clinical evidence‐based medicine data on respiratory‐digestive tract fistula, mostly from single‐center case studies, so it is difficult to form clinical diagnosis and management guidelines. In order to standardize the diagnosis and management of acquired respiratory‐digestive tract fistulas and summarize the achievements in recent years, we invite experts in related fields to form an expert committee to revise the expert consensus once again according to international research progress and Chinese clinical research by searching PubMed, Embase, Cochrane Library, China Journal Full‐text Database, Chinese Science and Technology Journal Database, and Wanfang full‐text Database. Considering that the digestive tract plays a leading role in the occurrence and development of the disease, the second edition consensus revised the name of the disease from “acquired respiratory‐digestive tract fistulas” to “acquired digestive‐respiratory tract fistulas”. The second edition consensus increased the treatment of acquired digestive‐respiratory tract fistulas after the esophageal operation, introduced the endoscopic vacuum negative pressure drainage technique, and further improved the clinical diagnosis and treatment plan. The implantation of the respiratory and digestive stent is the most important and best treatment for acquired digestive‐respiratory tract fistulas (ADRF). Chinese medicine experts are invited particularly to participate in replenishing the knowledge of traditional Chinese medicine and strive to fully reflect China's actual experience. After an online discussion, revision, and voting by the expert committee, a consensus has been reached.

## 2 REVISION PRINCIPLES

Under the guidance of the principle of evidence‐based medicine, the revision was carried out by referring to international norms, combined with national conditions, maneuverability, and new research data. The expert opinions were divided into five grades: grade 1 means complete agreement; grade 2 means agreement and opinions with reservations; grade 3 means opinions undecided; grade 4 means disagreement and grade 5 means complete disagreement. Those who voted for grades 2–5 were required to state the reasons and suggestions about how to improve the statement. When grade 1 and 2 opinions surpassed 80%, the consensus was reached.

## 3 ETIOLOGY, CLASSIFICATION, AND PATHOGENESIS OF ADRF

### 3.1 Etiology

#### 3.1.1 Benign disease^1^, ^2^

1. Traumatic: severe thoracic crush injury or trauma, esophageal foreign body, severe esophageal chemical burn.
2. Iatrogenic: long‐term compression of the tracheal tube with a balloon after the operation, esophageal stent implantation, and postoperative radiotherapy.
3. Infectious: tuberculosis infection of the lung, trachea, esophagus and mediastinal lymph nodes, esophageal, trachea/bronchial syphilis infection, and other causes of infection.
4. Spontaneous rupture of the esophagus
5. Structural abnormalities such as esophageal diverticulum
6. Crohn's disease, Behcet's disease, and other diseases involved in the esophagus.

#### 3.1.2 Malignant disease

ADRFs are more common in malignant lesions,^3^, ^4^ which often occur with advanced esophageal cancer (more than 70%,^5^, ^6^), advanced lung cancer, mediastinal malignant tumor, thyroid cancer, and other tumors metastasized to the respiratory tract and chest tumors after radiotherapy, chemotherapy, and immunotherapy.

### 3.2 Classification and pathogenesis

#### 3.2.1 Esophagorespiratory fistulas (ERF)

ERF refers to the pathological communication between the esophagus and respiratory tract. It is a serious life‐threatening complication with high mortality, low survival rate, and poor quality of life.^2^, ^7^, ^8^ ERF fistulas can occur anywhere in the trachea below the larynx and bilateral main bronchi, but the most common site is between the middle part of the esophagus and the left main bronchus.

Malignant ERF are mostly acquired from advanced esophageal cancer, and the formation mechanism includes^7^, ^9^:

1. Advanced esophageal cancer tissue invades the whole layer of the esophageal wall, and the upper and middle anterior wall of the esophagus is adjacent to the posterior wall of the trachea and left main bronchus. It is easy to form a fistula when the tumor involves the respiratory wall and grows too fast.
2. Radiotherapy not only kills tumor tissue but also damages the regeneration ability of normal esophageal tissue, resulting in tumor necrosis and low repair ability of normal tissue to form a fistula. At the same time, it leads to fibrosis stiffness of the esophageal wall and a decrease in contraction and relaxation function, which easily leads to fistula formation.
3. The absorption of tumor tissue necrosis after systemic chemotherapy can also lead to the formation of a fistula. Because of the local arterial infusion chemotherapy, tumor tissue necrosis is too fast, whereas normal tissue regeneration is slow, and a fistula can also be formed.
4. Dumbbell or trumpet‐shaped stents are often used to prevent the displacement of esophageal stents in the treatment of esophageal stricture in the late stage of esophageal cancer so that the two ends of the stents produce greater pressure or shear force on the esophageal wall, affecting the blood supply of the esophageal wall, especially the anterior wall. After the operation of esophageal cancer, the anastomotic stoma (arch, neck) recurred, and the residual esophagus and anastomotic stoma were implanted into the stent. The upper mouth of the straight tubular stent stimulates the curved normal esophageal wall and tracheal wall, and friction can easily cause tissue necrosis to form a fistula.

#### 3.2.2 Thoracostomach‐airway fistulas

After esophagogastrostomy or cervical anastomosis for esophageal cancer, there is a new adjacent relationship between the stomach and respiratory tract, and the fistula formed by the connection between the stomach and trachea or bronchi is one of the serious and life‐threatening complications after resection of esophageal cancer.^10^

The related factors for the formation of thoracostomach‐airway fistulas are:

1. Radiotherapy: If the tumor remains after the operation, the esophageal bed area will be treated with radiotherapy. The dose and tolerance dose of esophageal radiotherapy is 60–70 Gy, whereas the tolerance dose of the stomach is only half of that of the esophagus, which is about 30–40 Gy. Excessive radiation to the thoracic stomach in the esophageal bed area can easily lead to radiation gastric ulcer, gastric wall necrosis, perforation, and respiratory tract injury.
2. Gastric acid chemical stimulation and gastric juice digestive enzymes locally corrode gastric perforation, resulting in damage to the respiratory wall.
3. Pulmonary infection and local mediastinal inflammation.
4. Tumor recurrence and invasion.
5. Poor suture and local ischemia.
6. Chemotherapy.
7. Systemic malnutrition and so on.

#### 3.2.3 Esophageal anastomotic airway fistulas

The anastomosis between the esophagus and stomach is connected with the respiratory tract after the resection of esophageal cancer is a respiratory anastomotic fistula. After supra‐arch anastomosis after surgical resection of esophageal cancer, high‐dose radiotherapy in the anastomotic area or an anastomotic tumor recurrence and direct invasion of the respiratory tract can easily lead to anastomotic fistula. In addition, dilatation treatment for anastomotic stricture, anastomotic infection, and other conditions also easily leads to the formation of an anastomotic fistula.

#### 3.2.4 Esophago‐alveolar fistulas

A small number of patients form esophago‐alveolar fistula,^3^, ^11^ mainly originating from esophageal and bronchial carcinoma. Radiotherapy and chemotherapy lead to esophageal fistula, fistula destruction of the mediastinum, pleura and lung tissue, and promote the formation of an esophageal‐pleural‐alveolar fistula. All patients have aspiration pneumonia, and 79% of patients have pulmonary inflammation or abscess.^12^

## 4 DIAGNOSIS

By combining clinical symptoms, imaging examination, bronchoscopy, and gastroscopy, the diagnosis of ADRF is generally not difficult but more complex.

### 4.1 Clinical symptoms

The characteristic symptoms of ADRF are paroxysmal cough after swallowing, coughing up food residue, accompanied by persistent dysphagia and dyspnea and a large amount of white sticky sputum or bloody sputum and purulent sputum. Some patients have severe burning and irritant cough, aggravation of cough or cough in the supine position, relief or disappearance of cough in a sitting position, called “burning‐like cough syndrome.”

The clinical symptoms of thoracostomach‐airway fistula are more serious and dangerous than general ERF and esophageal anastomotic airway fistulas. Fasting can only reduce the entry of food into the respiratory tract, but a large number of digestive juices, such as gastric juice and bile, still flow into the respiratory tract through the fistula, which tends to expand rapidly in a short period of time because of the digestive effect of gastric juice. Patients will cough violently even if they do not eat, and the inflammation of the lungs is generally more serious, which is chemical inflammation in the early stage and is often associated with infectious inflammation such as bacteria and fungi in the later stage. When the fistula is large, because of a large amount of inhaled gas flowing into the gastric cavity, the patient has decreased respiratory function, respiratory failure and so on. If not treated in time, the patient will die quickly.

#### 4.1.1 Chinese traditional medicine point of view

Chinese traditional medicine believes that the disease belongs to the category of “cough” and “phlegm and drink.” The disease is due to cancer toxin erosion, difficulty in eating and other reasons, resulting in a deficiency of lung qi and spleen qi, and then leading to the deficiency of yang qi, deficiency of qi and blood, phlegm drink endogenous, choking respiratory tract, resulting in adverse lung qi, and finally developing a cough.

#### 4.1.2 TCM syndromes

Patients with the disease are always shown as excess in external, whereas the essence is a deficiency, which is mainly manifested as deficiency of lung and spleen qi, deficiency of yang qi of spleen and stomach, and the excess aspects mainly shown as turbid phlegm and drink. The following syndrome types are common in clinics:

1. Syndrome of cold drink invading lung: cough, coughing a large amount of white foam‐like sputum, aggravation when eating cold, chest tightness and shortness of breath, not thirsty, loose stool, fat tongue, slippery tongue coating, and slippery pulse.
2. Syndrome of phlegm turbidity obstructing the lung: coughing, spitting a large amount of white, lump sputum that is easy to spit out, with abdominal distension, loose stool, fat tongue, white and greasy tongue coating, and slippery pulse.
3. Deficiency of lung and spleen qi syndrome: sometimes coughing, with a small amount of phlegm, low and timid voice, sallow complexion, loose stool, fat tongue and thin tongue coating, and thin pulse.

### 4.2 Imaging examination

#### 4.2.1 Esophageal X‐ray radiography is of great value

40% meglumine diatrizoate should be selected as the contrast medium (iodine water contrast). Pressing the upper abdomen with hands during radiography can improve the diagnostic rate, but it is not the first choice for patients with ERF. Especially when the fistula is large, radiography should be cautious, and there may be serious aspiration when swallowing the contrast medium. As the contrast medium mistakenly into the respiratory tract is easy to cause severe cough, it is difficult to accurately evaluate the position, shape, length and diameter of the fistula during radiography, and sometimes it's difficult to show small fistulas.

Barium sulfate radiography is prohibited to prevent barium from entering the lungs through the fistula to form intractable foreign body‐deposited pneumonia.

In order to avoid blurring the image caused by a large number of bacteria in swallowing and severe choking, transoral esophagography or gastric tube radiography is recommended. Under imaging monitoring, the catheter is inserted into the predetermined fistula, 40% of the iodine water contrast agent is injected into the suspected esophageal fistula for about 3 mL, and the suspected gastric fistula is injected with about 5 mL contrast agent, which can avoid a large number of swallowing and choking, and a clearer image can be obtained.

#### 4.2.2 CT or MRI

CT and MRI are also sensitive methods for the diagnosis of ADRF, which can carefully observe the lesions of the respiratory tract, esophagus, chest, mediastinum, and stomach, which is very helpful in evaluating the degree of disease and pneumonia, clarifying the relationship between the fistula and surrounding tissue, and choosing the type and mode of stent implantation. In addition, for patients who need tracheal stents, CT reconstruction images can help to accurately measure the diameter of the respiratory tract and the distance between the fistula and the carina or glottis and facilitate the determination of the best stent specifications.

### 4.3 Endoscopy

#### 4.3.1 Bronchoscopy

The fistula, whether it is located in the trachea or bronchus, can be easily discovered by bronchoscopy. Suppose the fistula is very small and sometimes difficult to find. In that case, bronchoscopy after oral administration of methylene blue is helpful in finding the fistula, and it is also helpful to judge the existence of a small fistula by dynamically observing whether there are bubbles in the respiratory wall.

#### 4.3.2 Gastroscopy

It is also one of the important diagnostic means. It can look directly at the fistula or observe the bubbles from the fistula. It needs to be combined with esophagography to confirm the existence of the fistula. Gastroscopy can help observe the gastric mucosa around the fistula, and a biopsy can be performed if necessary to confirm the cause of the disease and help formulate treatment measures. Gastroscopy can also be used to observe the healing of the fistula after the airway stent is blocked.

## 5 TREATMENT

Patients with ADRF often have uncontrollable lung infections and severe malnutrition, and they may have a poor quality of life and generally deteriorate rapidly. Without active treatment in time, patients often die within a few days to months, with more than 90% of them dying from lung infections.

### 5.1 Surgical treatment

For patients with benign ADRF, the surgical resection of the fistula and pathological tissue should be done as far as possible.^13^ Pulmonary lobectomy or pneumonectomy is feasible for irreversible and pathological lung tissue. The esophageal diverticulum related to the fistula should also be removed, and trachea, bronchus, and esophageal defects should be sutured with double layers. Tissue such as pleura, muscle, pericardium or diaphragm flap can be placed between the esophagus and trachea to wrap the fistula to reduce recurrence. If there are many respiratory tract defects, it is feasible to transplant respiratory tract substitutes.

The general condition of patients of ADRF in esophageal surgery is poor, and it is difficult to tolerate re‐thoracotomy, and the incidence of reoperation fistula is still high. It is mainly treated in the clinic by adequate irrigation and drainage, anti‐infection, and fasting for solids and liquids. The placement of a jejunal nutrition tube to start enteral nutrition or intravenous parenteral nutrition support and other conservative treatment to create conditions for follow‐up surgery or interventional therapy. Long‐term ADRF occurs after esophageal surgery, and the scar tissue around the fistula proliferates obviously; if there is no evidence of malignant tumor recurrence or the fistula is located near the anastomosis, it can only be treated by endoscopy.

### 5.2 Interventional therapy

Interventional therapy guided by bronchoscope, gastroscope, and image is the main treatment of ADRF that is not suitable for operation, which can greatly reduce the symptoms of patients and improve their quality of life. At present, the most commonly used interventional therapy is the implantation of respiratory tract and/or digestive tract stents.^6^, ^7^, ^14^, ^15^, ^16^ Endoscopic metal clamp closure (such as over‐the‐scope), argon ion coagulation (APC) or electrocoagulation to promote the formation and closure of fistula granulation tissue, local spraying of fibrin glue, and local injection of tissue adhesive can also be used alone or in combination according to the situation. Recently, some studies have re‐evaluated the role of endoscopic vacuum negative pressure drainage in the closure of the fistula, showing a good effect. In addition, there are reports of successful closure of the fistula with cardiac interventricular septal/atrial septal umbrella occluder, and the curative effect needs to be further evaluated.

#### 5.2.1 Bracket

The ideal fistula closure stent should meet the following points:

1. Completely cover the fistula and fit well with the surrounding wall.
2. The stability of the implanted stent is good, especially stent coating is reliable and not easy to damage.
3. It is not easy to shift after stent implantation.
4. The stent can maintain a certain tension for a long time.
5. The recyclable stent should be placed in the benign fistula, whereas the stent should be implanted for a long time in malignant lesions.

##### Respiratory stent

###### Material selection

Respiratory tract‐covered metal stent or silicone stent can effectively reduce the flow of digestive tract secretions into the respiratory tract, reduce the inflow of respiratory gas into the digestive tract, and improve the quality of life of patients.

At present, the commonly used clinical covered metal stents include Ni–Ti alloy stent, Z‐shaped stainless steel covered metal stent, and Ultraflex stent.

###### Individual design of stent

**FIGURE 1:**
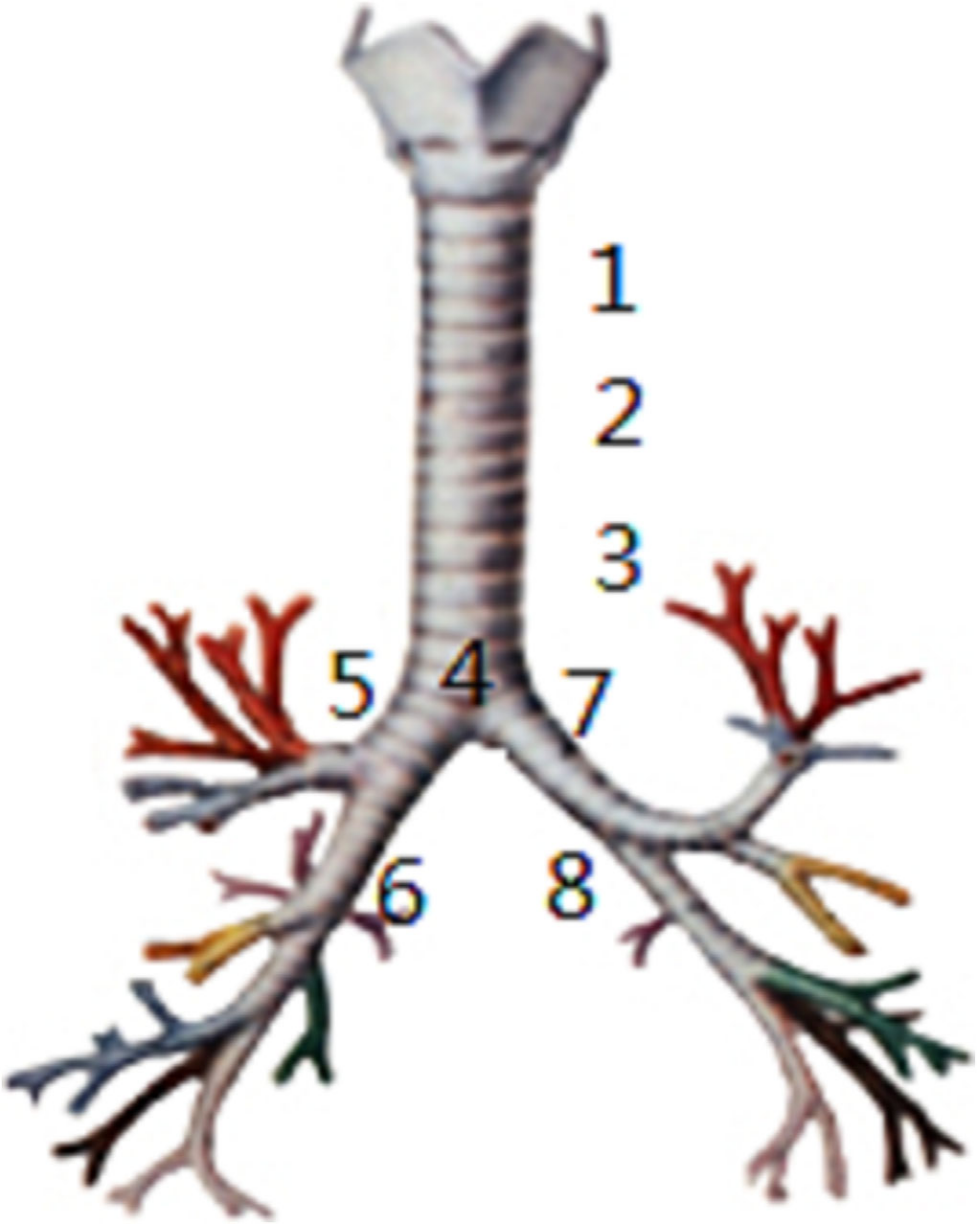
Schematic diagram of eight zones of the central respiratory tract.

1. The stent shape is selected according to the location of the fistula: The common straight stent (I shape) could be used when the fistula and the lesion lumen at least exceed the 10 mm normal lumen (such as zones I, VI, and VII), and the forked stent (Y or L shape) could be designed when the fistula was close to the carina (such as zones II, III, IV, V, and VII).^15^, ^17^ When there were not enough fixed sites above and below the fistula, the forked stent should be selected.^15^ The customizable small Y stent can be used for fistulas located in zones V, VI, VII, and VIII (Figure 1).
2. The stent length was determined according to the lesion length: The stent length at both ends should exceed the lesion range by 20 mm. For Ni–Ti shape memory alloy stent, the extension length of stent compression should be taken into account. When the fistula is large, the stent should cover each end of the fistula for more than 20 mm.
3. The diameter of the stent is determined according to the diameter of the upper and lower respiratory tract of the fistula and the degree of stenosis: The diameter is 10% to 20% larger than the inner diameter of the normal respiratory tract or equal to the anterior and posterior diameter of the respiratory tract generally, but for those with obvious stenosis near the fistula, it should be designed as a dumbbell type, flashlight type, or tube shape.
4. The measurement of respiratory tract length: Generally measured under a bronchoscope, the measurement value can be made more accurate using a positioning ruler or biopsy forceps and can also be calculated from the CT scanning level.
5. The measurement of respiratory tract diameter: The measurement on the fat window of thin CT at the focus during deep inhalation is generally more accurate, but the direct measurement is not accurate when the CT scan is the oblique section of the respiratory tract, so it needs to be converted according to the formula of Pythagorean law.

###### Stent placement method

The metal straight cylindrical stents of the respiratory tract are generally implanted under the guidance of the flexible bronchoscope, and the L‐shaped and Y‐shaped stents are best implanted under the rigid bronchoscope, or all the stents can be released under X‐ray fluoroscopy. The metal support conveyor is composed of a support conveying sheath with a guide (the inner core of the conveying sheath), an inner tube with a bracket, and an ejector rod behind the bracket; it is also called the three‐casing placement method. Release the bracket under the positioning ruler, the position is more accurate. In addition, silicone stents need to be placed under a rigid bronchoscope.

##### Esophageal stent

###### Esophageal stent materials

Most of the commonly used esophageal metal stents come from American Boston Scientific and Cook Endoscopy,^16^ South Korea S & G Biotech and Taewoong Medical companies,^6^ and domestic Ni–Ti memory alloy stents and Z‐type stainless steel stents. At present, the self‐expanding fully‐covered or partially‐covered metal stent with mushroom head at both ends is widely used, which is characterized in that it can effectively close the fistula. At the same time, it will prevent tumor and granulation tissue from blocking the stent cavity and reduce the incidence of stenosis after stent implantation. In addition, some esophageal stents with radioactive particles or chemotherapy sustained‐release particles can prevent tumor restenosis in patients with malignant ERF complicated with an esophageal stricture.

###### Individual design of esophageal stent

1. Design of ERF esophageal‐covered metal stent: The stent length should be at least 5 cm longer than the length of the fistula. After implantation, make sure that the upper edge of the stent (each end is longer than the fistula) is more than 2 cm higher than the upper edge of the fistula. The diameter of the stent is generally 17–20 mm. For those with a history of esophageal radiotherapy, the stent diameter is 14–16 mm. If the upper edge of the stent is to be placed near the entrance of the esophagus, the stent with a small diameter (15–17 mm) and no trumpet mouth should be selected.
2. Design of digestive tract stent for esophageal anastomotic airway tract fistula: Segmented fully‐covered esophageal and segmented mushroom fully‐covered stents are commonly used in clinics, and the diameter should not be too large; part of the stent is located in the esophagus, which is designed to refer to the esophageal stent. Part of it is located in the thoracic stomach, and its lower end can be designed in the shape of a large trumpet to reduce the phenomenon of lateral leakage caused by gastric contents entering the fistula between the outer surface of the stent and the gastric wall. In addition, it is better to add an anti‐reflux valve to the lower part of the stent to reduce the reflux of chest and stomach contents. If possible, it is best to place a respiratory stent.

###### Placement method

1. X‐ray‐guided implantation method: The advantage is that it can accurately judge the position of the guide wire, whether it passes through the lesion segment, and reduce iatrogenic perforation. It can dynamically monitor the stent release process, especially the release state of the lower edge of the stent.^18^ After the placement of the stent, the effect of fistula closure can be observed by radiography. X‐ray guidance alone cannot effectively (directly) observe esophageal lesions and fistula and real‐timely and effectively judge and deal with the complications such as bleeding and perforation in the process of stent implantation.
2. Gastroscope direct vision implantation method: Gastroscope implantation under direct vision has the advantages of simple operation, high success rate, avoiding X‐ray (radiation) injury, timely treatment of intraoperative bleeding, making the implantation process safer, and the stent position can be adjusted in time under direct vision. However, problems such as poor positioning accuracy are easy to occur when stents are implanted under a gastroscope.

##### Selection of stents for different digestive‐respiratory tract fistulas

###### ERF

###### Single esophageal stents

Esophageal stents alone can be used to close fistulas in malignant and some benign ERF patients without surgical indications.^19^ European Society of Gastrointestinal Endoscopy (ESGE) recommended that esophageal self‐expanding covered metal stent implantation is the first choice for the treatment of malignant ERF. Esophageal stent implantation be considered for benign ERF, but specific types of stents are not recommended. The best time for stent retention should be individualized.^16^ When the patient has esophageal stricture, without or with mild respiratory stricture, operators can choose a single esophageal stent. For ERF patients with no obvious esophageal stricture, leading to a high displacement rate of esophageal covered stents alone, we can consider the implantation of esophageal non‐covered stents combined with covered bimetallic stents.^20^ The former plays a fixed role, and the latter plays a role in blocking the fistula.

With the improvement of the technology of covered metal stents and the improvement of digestive endoscopy, upper esophageal ERF can also be treated by stent implantation. Some domestic studies have considered that the high fistula, whose upper edge of the stent does not exceed the upper edge of the first physiological stricture of the cervical segment of the esophagus, is still an indication. The long‐term effect is satisfactory, and the complications have not increased.^21^

##### Single respiratory tract stent

1. If the ERF located in the upper cervical esophagus is unable to place the esophageal stent, tracheal stent implantation may be considered.
2. If the esophageal lumen at the distal end of the fistula is completely blocked, and the guide wire cannot be successfully inserted into the gastric cavity, the esophageal stent cannot be placed.
3. Esophageal stent implantation can easily lead to esophageal rupture.
4. There is moderate to severe respiratory stenosis, and there is no or mild esophageal stricture. Stent placement in the respiratory tract can not only relieve the stenosis but also block the fistula.

##### Combined implantation of the respiratory stent

1. If the effect of esophageal stent implantation is not good, tracheal stents may be considered at this point, and tracheal stents can be considered and removed if necessary.^16^, ^22^
2. When moderate to severe strictures of the esophagus and central respiratory tract are involved, as the insertion of a single stent may not be sufficient to relieve symptoms, the combined implantation of esophageal and tracheal stents can be considered.^23^ In this case, the respiratory stent should be placed first, and then the esophageal stent should be placed. Therefore, it is necessary to systematically and comprehensively evaluate ERF patients before the combined implantation of esophageal and respiratory stents. Pay attention to the size of the fistula and its relationship with blood vessels. If the fistula is too close to the large blood vessel, use the stent carefully.
3. Patients with an easy displacement of esophageal stent: Through the placement of a respiratory stent, the interaction between the respiratory and esophageal stents makes it difficult to move the esophageal stent.

###### Respiratory‐thoracogastric fistulas

If the fistula is located in the broad gastric body, stent implantation through the esophagogastric approach cannot block the fistula, only a respiratory stent can be used to block the fistula, and the bifurcated respiratory stent is needed in most cases. However, if it occurs in the tubular stomach, esophageal stents can be used to block it.

###### Esophageal anastomosis airway fistulas

A respiratory stent should be first implanted, and a special esophageal stent can be made if necessary. Because of the special position of the esophagus anastomosis, it is difficult to completely block the fistula with the digestive tract stent, which can only reduce the inflow of food and secretions into the respiratory tract.

###### Esophago‐alveolar fistulas

Benign or resectable malignant esophago‐alveolar fistula should be cured as far as possible. Endoscopic interventional therapy needs to grasp the indications. Interventional therapy will affect drainage and aggravate pulmonary infection. Generally, pulmonary infection is not serious; patients with a fistula within 1 cm can choose a metal clip or over‐the‐scope clip (OTSC), local burning. The esophageal stent can be used as preoperative transitional treatment. Patients with malignant esophago‐alveolar fistula can choose esophageal stent implantation without surgical conditions, and patients with poor conditions can choose conservative medical treatment such as enteral nutrition.

##### Evaluation of curative effect

Clinical practice has proved that airway and esophageal stent implantation has the advantages of less operative trauma, fewer complications, and definite curative effect. The operation is not limited by the patient's physical condition and age, and the stent can be removed at any time, which has become the most feasible and commonly used method for the palliative treatment of ADRF. The ideal short‐term effect after stent implantation should be tight closure of the fistula, no gas and liquid in and out of the fistula, tolerance of foreign body sensation in the esophagus and airway, no complications such as stent displacement, bleeding, and asphyxia, and no contrast overflow in angiography. The ideal long‐term effect is that the tissue around the fistula grows to cover the fistula, and the infection disappears, and some patients can be removed by tracheal stents, especially benign airway fistulas.

At present, there is no unified standard for judging the curative effect of respiratory fistula at home and abroad. Professor Hongwu Wang established the standard for judging the efficacy of stent fistula closure based on his own experience^15^: (1) Complete relief of (CR), fistula healing, complete relief of clinical symptoms (such as drinking water, cough, fever, etc.) lasted for 1 month; (2) clinical complete remission (CCR), fistula does not heal, but is completely blocked by stents, clinical symptoms are completely relieved for 1 month; (3) partial remission of (PR), fistula is not closed, partly occluded by stents, and clinical symptoms are partially relieved. (4) The fistula of ineffective (NR) fistula is not closed, and it is not blocked by stents, and the clinical symptoms are not relieved.

The analysis of the possible factors of lax stent occlusion is as follows^17^:

1. The stent is not in shape, and the airway shape and diameter are slightly longer and thinner when inhaling and then restored when exhaling, so it may be due to the exhalation phase of the airway during CT image acquisition; the stent design is too small, so the fistula closure of patients is not tight.
2. The placement of the stent was not accurate, so the stent did not reach the expected position of the patient before the operation, so the occlusion effect was not as expected.
3. In patients with esophageal airway malignant fistula with esophageal stricture, the poor opening of the esophageal stent may result in lax adherence of the esophageal stent when blocking the side of the esophagus, which makes the contrast overflow through the gap between the edge of the proximal stent and the esophageal wall, which is the so‐called funnel phenomenon.

In view of the above adverse factors, the solutions are as follows:

1. Inspiratory phase CT examination and accurate measurement of airway diameter, 3D printing stent if necessary.
2. The placement of an airway stent under general anesthesia can locate the stent accurately during the operation.
3. Patients should not eat and drink within 3 days, and a small amount of secretion between the stent and the tube wall can effectively seal the fistula.

At present, there is no evidence‐based medical data to support the clinical efficacy of benign ERF. Based on several existing retrospective studies, the success rate of various intervention methods in the treatment of benign ERF is nearly 95%, and the success rate of continuous clinical remission is more than 90%. A number of retrospective studies have shown that the success rate of fistula closure with self‐expanding covered metal stents in the treatment of malignant ERF is 70%–100%.^6^, ^9^, ^24^, ^25^, ^26^, ^27^, ^28^

##### Complications

The early and long‐term complications of stent implantation in patients with ADRF. At present, the data on evidence‐based medicine are mostly from individual or retrospective case reports.

###### Tracheal stent

The main complications of stent implantation are cough, secretion retention, lax fistula blockage, granulation tissue hyperplasia, pulmonary infection, and so on. Metal stents may also have complications such as stent displacement or prolapse, halitosis, metal fatigue, stent fracture, respiratory tract infection, tracheobronchial wall perforation, massive bleeding, and so on. These complications are generally non‐fatal and manageable.^29^

###### Esophageal stent

The incidence of operation‐related complications is about 0%–27%, and the mortality rate is 0%–12%. Complications include fistula reopening, stent blockage caused by tumor tissue growth or food, stent displacement, stent membrane destruction and pain, dysphagia, foreign body sensation, bleeding, and pneumonia.^6^, ^27^, ^30^, ^31^ Based on some retrospective studies, it was reported that 1 month after stent implantation, 10% to 30% of ERF patients with successful initial treatment had the problem of fistula reopening.^7^ Endoscopic stent adjustment or re‐stent implantation can solve the problems of incomplete fistula closure, stent blockage, and displacement.

##### Postoperative management and follow‐up

Respiratory tract or esophageal stent should both be strictly managed and regularly followed up after the operation, and timely treatment should be made if complications occur. It is easy to have secretion retention within 2 weeks after a respiratory stent operation. The alkaline liquid should be inhaled by ultrasonic nebulization at least 4–6 times a day, combined with intravenous fluid replacement and humidification of sputum, so that sputum is easy to cough up, and the bronchoscope should be reexamined once a week. Granuloma is easy to occur after 1 month, and the bronchoscope should be reexamined once a month within 3 months. After half a year, the membrane of some metal‐coated stents may break, and the symptoms of choking will appear again, so the stents need to be replaced in time. The esophageal stent is easy to shift in the early stage, so it is necessary to observe closely and adjust the position of the stent in time. Granulation tissue is easy to appear at both ends of the stent after 3 months, and the blockage should be dealt with in time.

#### 5.2.2 Other intervention measures of the digestive endoscope

##### OTSC

The application of OTSC has been gradually increased in the treatment of ERF. There are many reports about the successful clipping of benign traumatic ERF by OTSC in China.^32^, ^33^ OTSC anastomosis clip can be divided into two types: blunt tooth shape and cusp tooth shape; cusp tooth shape is suitable for fibrosis, ulcer, and fistula, and blunt tooth shape is suitable for closing non‐fibrotic tissue. The common specifications of OTSC are 11, 12, and 14 mm, which should be selected according to the size of the fistula. OTSC is generally suitable for the early stage of fistula formation, the tissue fibrosis around the fistula is not severe, and the diameter of the fistula is less than 3 cm. The specific operation is to install the OTSC on the outside of the front of the gastroscope, align the front transparent cap with the fistula and attract the fistula under full negative pressure, inhale the fistula and the surrounding tissue into the clamp cap and grasp the tissue around the fistula with both arms grasping forceps, and then release OTSC to close the fistula. The application of OTSC in the esophagus is more difficult than that in the stomach. The influencing factors include the small lumen of the esophagus and the parallel angle between the esophageal wall and the OTSC operating system. OTSC closure indications and model selection for different types of ERF still need large samples and high‐quality clinical data support. Generally speaking, sharp‐tooth OTSC can be used to close the fistula for early and benign ERF caused by trauma or iatrogenic. The marginal tissue fibrosis of chronic benign ERF fistula or the tissue around malignant ERF fistula is brittle, and it is difficult to close OTSC, which is easy to cause the anastomotic clip to fall off or cannot be closed. Of course, there are also reports of successful closure of chronic ERF with OTSC. In addition, whether APC and other methods are needed to facilitate granulation tissue formation to improve the closure effect before OTSC closure of the severe fibrotic fistula, the point of view is not unified, which needs to be further studied.

##### Endoscopic vacuum negative pressure drainage technique (EVT)

EVT may play a better effect in the closure of the fistula. EVT originates from vacuum‐assisted wound healing technology. The sponge in contact with the wound needs to be molded and placed into the fistula according to the condition of the fistula, then the sponge should be sewn at the front of the nasal/percutaneous drainage tube, and the side hole of the drainage tube should be embedded into the sponge to ensure smooth drainage. Meta‐analysis re‐evaluated the efficacy of EVT in the treatment of ERF. It was significantly better than stent implantation in fistula closure rate, with lower complications and mortality, but more endoscopic interventions (such as repeated replacement of drainage sponges, etc.) and long‐term hospitalization.

#### 5.2.3 Other techniques of tracheal endoscopy

Tracheal stent implantation combined with tracheal endoscopic cauterization and local perfusion closure of biological glue fistula are suitable for the treatment of small fistula or combined with a stent, but the fistula will be recanalized 1 or 2 weeks after occlusion, so the clinical application is less.^34^

Stem cell injection under bronchoscopy may have a good effect. Early studies based on animal experiments confirmed that bone marrow mesenchymal stem cells could repair the bronchopleural fistula.^35^ Based on human studies, it is reported that bone marrow mesenchymal stem cells were injected into a patient with a right main bronchopleural fistula after right pneumonectomy (the size of the fistula was about 3 mm).^36^ Two months after the operation, the fistula was completely closed, and the pathology of the biopsy at the fistula showed that there was a proliferation of respiratory epithelial cells in the upper layer of the fibrous lamina propria, whereas smooth muscle cells decreased and were replaced by fibroblasts. Immunohistochemistry showed the phenotype of p40 and DNp63, suggesting that the basal cells differentiated into squamous epithelium, showing a good repair effect. In addition to bone marrow mesenchymal stem cells, there are also reports of successful treatment of patients with bronchopleural fistula and bronchomediastinal fistula by local injection of adipose stem cells under bronchoscope.^37^, ^38^

### 5.3 Conservative treatment in internal medicine

Medical conservative treatment is the basic treatment for patients with ADRF whose general condition is poor and cannot tolerate surgery, including the use of antibiotics to control pulmonary infection, high intravenous nutrition, jejunostomy and other supportive treatment, and symptomatic treatment such as diluting sputum relieving cough. In addition, for patients with thoracostomach‐airway fistula and esophageal anastomotic airway fistula, in addition to fasting, gastrointestinal decompression should be carried out to reduce the flow of acidic gastric juice into the respiratory tract.

#### 5.3.1 Anti‐infection

Domestic studies have reported that the etiological culture of the lower respiratory tract of ERF patients is mainly gram‐negative bacteria and fungi, accounting for 64.7% and 25.5%, respectively, of which *Pseudomonas aeruginosa* is the most common, which may be related to the repeated use of antibiotics and intestinal flora translocation.^39^ Sensitive antibiotics should be selected in the treatment, the curative effect should be evaluated, and the medication should be adjusted in time.

#### 5.3.2 Nutritional support

ERF patients often suffer from severe malnutrition due to the inability to eat by mouth and the stress and inflammation caused by infection. Malnutrition leads to delayed or non‐healing of the fistula and a significant increase in clinical complications. Therefore, active and effective nutritional support is very important for the overall therapeutic effect of ERF.^40^, ^41^, ^42^, ^43^

After a comprehensive evaluation of the nutritional status of the patients, a nutritional treatment plan was made. Enteral nutrition pathway should be established as soon as possible; a jejunal nutrition tube should be recommended; during the transition from parenteral nutrition to enteral nutrition, the overall daily nutrient requirement should be met. It is recommended to use a continuous micro pump to control the speed of the pump into the enteral nutrition solution, which can be accelerated gradually from the low speed. Energy is given at 25–35 kcal/(kg·d). The protein starts with 1 g/(kg·d), and the target supply is 1.2 ~ 2 g/(kg·d). When the intestinal function is normal, whole protein enteral nutrition preparation is recommended, and when the intestinal function is poor, elemental enteral nutrition preparation can be selected. Supply trace elements according to the recommended intake of nutrients and adjust them according to the monitoring results. It is necessary to regularly evaluate the effectiveness of nutritional treatment.

#### 5.3.3 Traditional Chinese medicine treatment

The treatment aims at its pathogenesis, which can supply symptomatic treatment in acute conditions or radical treatment in chronic conditions. We can also choose to strengthen vital qi to eliminate a pathogenic factor or eliminate a pathogen first or supply reinforcement and elimination in combination. According to different clinical syndrome types, different schemes are adopted:

1. Syndrome of cold drink invading lung: It is treated with a warming yang for dispelling cold drinks, relieving cough, and lowering adverse qi. Lingganwuweijiangxin decoction, licorice‐dried ginger decoction, or Xiaoqinglong decoction can be used to cure the disease.
2. Syndrome of phlegm turbidity obstructing the lung: It is treated by eliminating dampness and phlegm, lowering adverse qi and relieving cough. Er Chen decoction or Sanziyangqin decoction can be chosen.
3. Deficiency of lung and spleen qi syndrome: It is treated with a tonifying middle qi, invigorating spleen, and resolving phlegm. Choose Liujunzi decoction or Buzhongyiqi decoction.

### 5.4 Prognosis

The prognosis of the patients is significantly related to the stage of the disease and the method of treatment.

The significance of stent implantation in the treatment of survival in patients with ADRF is inconclusive. After esophageal and/or respiratory stent implantation, the survival time of patients with malignant ERF depends on the closure of the fistula. If the fistula can be effectively closed and pulmonary infection can be controlled, the quality of life and survival time of patients can be significantly improved. A controlled study showed that the quality of life of the patients with malignant ERF in the stent implantation group was significantly better than that in the control group and gastrostomy group,^16^, ^44^ especially in the improvement of dyspnea, dysphagia, dietary problems, dry mouth, cough and excessive saliva secretion, and the survival time increased significantly. A series of case studies reported that the average survival time of the stent implantation group was 3.4 months, which was significantly higher than that of the enterostomy and simple nutritional support groups.^28^

Studies^45^, ^46^ have shown that the median survival time of esophageal squamous cell carcinoma patients with esophageal fistula is 8.00–11.00 months, and the median survival time after fistula is 2.50–3.63 months. Sex, tumor treatment before fistula, type of esophageal fistula, treatment of esophageal fistula, and hemoglobin level significantly affect the prognosis of the disease. Among them, sex, type of esophageal fistula, and hemoglobin level are independent influencing factors, whereas esophageal stent implantation is a protective factor affecting prognosis.

## AUTHOR CONTRIBUTIONS

Hongwu Wang, Wen Li, and Zikai Wang wrote the consensus; other experts were involved in the formulation of this consensus.

## CONFLICT OF INTEREST STATEMENT

No authors report any conflict of interest.

## ETHICS STATEMENT

This paper is a consensus of clinical diagnosis and treatment and does not involve ethical approval. All the authors agree to publish.

## Data Availability

Data sharing not applicable to this article as no datasets were generated or analysed during the current study.

## References

[1] van Halsema EE , van Hooft JE . Clinical outcomes of self‐expandable stent placement for benign esophageal diseases: a pooled analysis of the literature. World J Gastrointest Endosc. 2015;7(2):135‐153. doi:10.4253/wjge.v7.i2.135 25685270 PMC4325310

[2] Lenz CJ , Bick BL , Katzka D , et al. Esophagorespiratory fistulas: survival and outcomes of treatment. J Clin Gastroenterol. 2018 Feb;52(2):131‐136. doi:10.1097/MCG.0000000000000751 27824640

[3] Martini N , Goodner JT , D'Angio GJ , Beattie EJ Jr . Tracheoesophageal fistula due to cancer. J Thorac Cardiovasc Surg. 1970;59(3):319‐324. doi:10.1016/S0022-5223(19)42464-1 4190046

[4] Balazs A , Galambos Z , Kupcsulik PK . Characteristics of esophagorespiratory fistulas resulting from esophageal cancers: a single‐center study on 243 cases in a 20‐year period. World J Surg. 2009;33(5):994‐1001. doi:10.1007/s00268-009-9988-3 19288038

[5] Marczyński W , Pająk M , Komandowska T , Nikiel I . Self‐expandable metallic stents in oesophago‐respiratory fistulas treatment in neoplasms ‐ case reports and literature review. Pneumonol Alergol Pol. 2015;83(4):303‐306. doi:10.5603/PiAP.2015.0050 26166792

[6] Shin JH , Kim JH , Song HY . Interventional management of esophagorespiratory fistula. Korean J Radiol. 2010;11(2):133‐140. doi:10.3348/kjr.2010.11.2.133 20191059 PMC2827775

[7] Shin JH , Song HY , Ko GY , Lim JO , Yoon HK , Sung KB . Esophagorespiratory fistula: long‐term results of palliative treatment with covered expandable metallic stents in 61 patients. Radiology. 2004;232(1):252‐259. doi:10.1148/radiol.2321030733 15166325

[8] Freitag L . Treatment of airway‐esophageal fistulas; 2013:421‐434.

[9] Murthy S , Gonzalez‐Stawinski GV , Rozas MS , Gildea TR , Dumot JA . Palliation of malignant aerodigestive fistulae with self‐expanding metallic stents. Dis Esophagus. 2007;20(5):386‐389. doi:10.1111/j.1442-2050.2007.00689.x 17760651

[10] Han X , Wu G , Ma N , et al. Clinical and imaging diagnosis of thoracic gastrotracheal (main bronchial) fistula. J Zhengzhou Univ (Medical Edition). 2003;3:395‐397. (in Chinese).

[11] Duranceau A , Jamieson GG . Malignant tracheoesophageal fistula. Ann Thorac Surg. 1984;37(4):346‐354. doi:10.1016/S0003-4975(10)60745-X 6201144

[12] Kim KR , Shin JH , Song HY , et al. Palliative treatment of malignant esophagopulmonary fistulas with covered expandable metallic stents. AJR am J Roentgenol. 2009;193(4):W278‐W282. doi:10.2214/AJR.08.2176 19770295

[13] Seto Y , Yamada K , Fukuda T , et al. Esophageal bypass using a gastric tube and a cardiostomy for malignant esophagorespiratory fistula. Am J Surg. 2007;193(6):792‐793. doi:10.1016/j.amjsurg.2006.07.023 17512299

[14] Melendez J , Chu D , Bakaeen FG , Casal RF . Tracheoesophageal fistula due to migration of a self‐expanding esophageal stent successfully treated with a silicone "Y" tracheobronchial stent. J Thorac Cardiovasc Surg. 2011;141(6):e43‐e44. doi:10.1016/j.jtcvs.2011.02.002 21429528

[15] Wang H , Tao M , Zhang N , et al. Airway covered metallic stent based on different fistula location and size in malignant tracheoesophageal fistula. Am J Med Sci. 2015;350(5):364‐368. doi:10.1097/MAJ.0000000000000565 26422803

[16] Spaander MCW , van der Bogt RD , Baron TH , et al. Esophageal stenting for benign and malignant disease: European Society of Gastrointestinal Endoscopy (ESGE) guideline ‐ update 2021. Endoscopy. 2021;53(7):751‐762. doi:10.1055/a-1475-0063 33930932

[17] Zhao C , Xiang S , Su W , et al. Clinical application of individualized metal‐coated stent in the treatment of airway fistula. J Interv Radiol. 2019;28(12):1185‐1189. (in Chinese).

[18] Xie Y , Zhang J , Chen Q . Clinical application of stent placement in esophagotracheal fistula with different causes under DSA localization. Anhui Med. 2014;35(3):315‐317. (in Chinese).

[19] Yang D , Ma H , Zhang X , et al. New progress in the treatment of esophageal fistula. Huaxi Med. 2015;30(10):1983‐1985. (in Chinese).

[20] Ma H , Zou H , Zhang J , et al. Clinical study of esophageal double stent implantation in the treatment of esophagotracheal fistula. Basic Med Clinic. 2015;35(7):968‐971. (in Chinese).

[21] Yang Y , Pang Z , Liu X . Clinical evaluation of endoscopic stent implantation in the treatment of 31 cases of upper esophageal carcinomatous esophagotracheal fistula. Gastroenterology. 2004;6:377‐378. (in Chinese).

[22] Wu B , Mao A , Wu S . Endoscopic direct vision combined with X‐ray guided interventional treatment of esophagotracheal fistula: a case report. J Interventional Radiol. 2013;22(7):615‐616. (in Chinese).

[23] Li Y , Ke M , Wu X , et al. Tracheoesophageal double stents in the treatment of 55 cases of malignant esophagotracheal fistula complicated with tracheal stenosis. J Practical Med. 2016;32(11):1847‐1849. (in Chinese).

[24] Saxon RR , Barton RE , Katon RM , et al. Treatment of malignant esophageal obstructions with covered metallic Z stents: long‐term results in 52 patients. J Vasc Interv Radiol. 1995;6(5):747‐754. doi:10.1016/S1051-0443(95)71180-0 8541679

[25] Saxon RR , Barton RE , Katon RM , et al. Treatment of malignant esophagorespiratory fistulas with silicone‐covered metallic Z stents. J Vasc Interv Radiol. 1995;6(2):237‐242. doi:10.1016/S1051-0443(95)71104-6 7540442

[26] Abadal JM , Echenagusia A , Simo G , Camuñez F . T treatment of malignant esophagorespiratory fistulas with covered stents. Abdom Imaging. 2001;26(6):565‐569. doi:10.1007/s002610000193 11911165

[27] Kishi K , Nakao T , Goto H , et al. A fast placement technique for covered tracheobronchial stents in patients with complicated esophagorespiratory fistulas. Cardiovasc Intervent Radiol. 2005;28(4):485‐489. doi:10.1007/s00270-003-0203-x 16010516

[28] Balazs A , Kupcsulik PK , Galambos Z . Esophagorespiratory fistulas of tumorous origin. Non‐operative management of 264 cases in a 20‐year period. Eur J Cardiothorac Surg. 2008;34(5):1103‐1107. doi:10.1016/j.ejcts.2008.06.025 18678504

[29] Ke MY , Huang R , Lin LC , Zeng JL , Wu XM . Efficacy of the Dumon™ stent in the treatment of airway gastric fistula: a case series involving 16 patients. Chin Med J (Engl). 2017;130(17):2119‐2120. doi:10.4103/0366-6999.213420 28836558 PMC5586184

[30] Binkert CA , Petersen BD . Two fatal complications after parallel tracheal‐esophageal stenting. Cardiovasc Intervent Radiol. 2002;25(2):144‐147. doi:10.1007/s00270-001-0088-5 11901435

[31] Nam DH , Shin JH , Song HY , Jung GS , Han YM . Malignant esophageal‐tracheobronchial strictures: parallel placement of covered retrievable expandable nitinol stents. Acta Radiol. 2006;47(1):3‐9. doi:10.1080/02841850500334989 16498926

[32] Ye M , Zhou Y , He Y , et al. One case of traumatic esophagotracheal fistula was clipped by OTSC anastomosis system under endoscope. Chin J Endosc. 2016;22(3):108‐110. (in Chinese).

[33] Yang W , Rao G , Ban J , et al. Endoscopic closure of OTSC system for the treatment of esophageal fistula: a report of 3 cases. Minimally Invasive Med. 2017;12(2):272‐273. (in Chinese).

[34] Liu S , Wang J , Liu Z , et al. 32 cases of esophagotracheal fistula treated with fibrin glue under gastroscope. Chin J Dig Endosc. 2010;04:215‐216. (in Chinese).

[35] Petrella F , Toffalorio F , Brizzola S , et al. Stem cell transplantation effectively occludes bronchopleural fistula in an animal model. Ann Thorac Surg. 2014;97(2):480‐483. doi:10.1016/j.athoracsur.2013.10.032 24370201

[36] Petrella F , Spaggiari L , Acocella F , et al. Airway fistula closure after stem‐cell infusion. N Engl J Med. 2015;372(1):96‐97. doi:10.1056/NEJMc1411374 25551543

[37] Alvarez PD , García‐Arranz M , Georgiev‐Hristov T , et al. A new bronchoscopic treatment of tracheomediastinal fistula using autologous adipose‐derived stem cells. Thorax. 2008;63(4):374‐376. doi:10.1136/thx.2007.083857 18364447

[38] Díaz‐Agero Álvarez PJ , Bellido‐Reyes YA , Sánchez‐Girón JG , García‐Olmo D , García‐Arranz M . Novel bronchoscopic treatment for bronchopleural fistula using adipose‐derived stromal cells. Cytotherapy. 2016;18(1):36‐40. doi:10.1016/j.jcyt.2015.10.003 26552766

[39] Gao Y , Wang H , Zhou Y , et al. Etiological characteristics of esophagotracheal fistula complicated with lower respiratory tract infection. Int Respir J. 2017;37(3):171‐172. (in Chinese).

[40] Huang H , Guo T , Cheng G . Clinical analysis of 63 cases of esophageal carcinoma complicated with tracheoesophageal fistula. Hainan Med. 2013;24(24):3624‐3626. (in Chinese).

[41] Silon B , Siddiqui AA , Taylor LJ , Arastu S , Soomro A , Adler DG . Endoscopic Management of Esophagorespiratory Fistulas: a multicenter retrospective study of techniques and outcomes. Dig Dis Sci. 2017;62(2):424‐431. doi:10.1007/s10620-016-4390-0 28012101

[42] Persson S , Rouvelas I , Irino T , Lundell L . Outcomes following the main treatment options in patients with a leaking esophagus: a systematic literature review. Dis Esophagus. 2017;30(12):1‐10. doi:10.1093/dote/dox108 28881894

[43] Arends J , Bachmann P , Baracos V , et al. ESPEN guidelines on nutrition in cancer patients. Clin Nutr. 2017;36(1):11‐48. doi:10.1016/j.clnu.2016.07.015 27637832

[44] Hu Y , Zhao YF , Chen LQ , et al. Comparative study of different treatments for malignant tracheoesophageal/bronchoesophageal fistulae. Dis Esophagus. 2009;22(6):526‐531. doi:10.1111/j.1442-2050.2009.00950.x 19302211

[45] Guan X , Liu C , Zhou T , et al. Survival and prognostic factors of patients with esophageal fistula in advanced esophageal squamous cell carcinoma. Biosci Rep. 2020;40(1). doi:10.1042/BSR20193379 PMC6960064 31894852

[46] Kawakami T , Tsushima T , Omae K , et al. Risk factors for esophageal fistula in thoracic esophageal squamous cell carcinoma invading adjacent organs treated with definitive chemoradiotherapy: a monocentric case‐control study. BMC Cancer. 2018;18(1):573. doi:10.1186/s12885-018-4486-3 29776344 PMC5960135

